# Top 100 cited articles in the thromboangiitis obliterans: a bibliometric analysis and visualized study

**DOI:** 10.1186/s40001-023-01540-6

**Published:** 2023-12-02

**Authors:** Zhenxing Liu, Weiwei Ning, Jinlong Liang, Tao Zhang, Qingxu Yang, Jie Zhang, Ming Xie

**Affiliations:** Department of Gastrointestinal Surgery, Affiliated Hospital of Zunyi Medical University, Zunyi, Guizhou China

**Keywords:** Thromboangiitis obliterans, Buerger's disease, Limb ischemia, Bibliometric, Visualized study, Top cited articles

## Abstract

**Objective:**

Thromboangiitis obliterans (TAO) is one of the most common types of peripheral arterial disease (PAD). This study aimed to explore the characteristics of the top 100 most cited articles in the TAO.

**Methods:**

A bibliometric analysis based on the Web of Science (WOS) database was performed. Literature was retrieved and ranked by the citations. Listed below are the top 100 citations, including original articles, reviews, full-length proceeding papers, and case reports that were included for analysis. The type of literature, research areas, and languages were recorded. The trends of citations including the total citations, an analysis of publication and citation numbers were conducted each year. We analyzed citations from highly cited countries, authors, institutions, and journals. Research hotspots were gathered by a visualized analysis of author keywords.

**Results:**

Most of the highly cited literature was original articles. A rising trend was observed in the number of citations per year. The peaks in the number of highly cited articles appeared in the year 1998 and 2006. The majority of the articles focused on the cardiovascular system and surgery. Journal of Vascular Surgery published most of the highly cited articles. The USA and Japan contributed nearly half the number of highly cited articles. Mayo Clinic and Nagoya University were highly cited institutions. Shionoya S and Olin JW were both the author with the largest number of citations and the most highly cited author in the reference. Articles that were highly cited most often addressed the following topics: “vasculitis”, “autoimmune disease”, and “critical limb ischemia”. Keywords that were mostly used in recent years were “stem cell therapy”, “progenitor therapy”, and “immunoadsorption”. The detection of bursts of author keywords showed the following: “permeability”, “differentiation”, and “critical limb ischemia” are recent keywords that have burst.

**Conclusions:**

In this study, the highly cited contributors in the field of TAO research were identified. Most cited articles in the top 100 focused on the cardiovascular system and surgery. Treatment and pathophysiology including stem cell therapy, progenitor therapy, genetics, autoimmunity, and inflammation are the hotspots of TAO.

**Supplementary Information:**

The online version contains supplementary material available at 10.1186/s40001-023-01540-6.

## Introduction

Thromboangiitis obliterans (TAO), also called Buerger's disease, is one of the peripheral arterial diseases (PAD) that manifests as limb ischemia, pain, intermittent claudication, weakened or disappeared dorsal artery pulse, migratory superficial phlebitis, and extremity ulcers and necrosis in severe cases [1, 2]. From a global perspective, the high-incidence areas of thromboangiitis obliterans are concentrated in the Middle East, Siberia, Japan, and South Korea. However, it is rare in America, Western and Northern Europe [3–5].

The specific etiology of TAO remains unknown, but it has been demonstrated that it mainly affects young males and is strongly related to smoking. It is generally believed to be the result of a combination of multiple factors, the potential mechanism of TAO includes the history of exposure to cold, smoking, autoimmunity, genetic factors including ethnic aggregation and genetic predisposition, oxidative stress, and inflammation, hemodynamic changes like hypercoagulable state of blood, and even periodontal diseases [2, 6, 7]. Other findings about the factors associated with the development of TAO include antiphospholipid antibodies, protein S deficiency, and platelet hyperreactivity to serotonin. However, it has been demonstrated that these mechanisms were not essential for the pathology of TAO [8]. In the early stage of the disease, whether thrombosis or inflammation promotes the progress of the disease is controversial. Thus it is necessary to investigate the highly concerned research areas in the field of TAO.

The management of TAO also needs to be further improved as the progress in the research of pathology and risk factors. Including smoking cessation, medicine, endovascular surgery, thrombolysis, radiofrequency ablation, transplantation of autologous Bone marrow-derived stem cells, and novel therapies [9–11]. The surgical treatment of TAO can be combined with autologous cell therapy and other novel therapies that trigger inflammations and autoimmunity [6, 12]. The challenges in the management of TAO reflect an urgency to reveal the hotspots of TAO research, which can be revealed by analyzing the characteristics of the highly cited articles. Thus, it is necessary to conduct a bibliometric analysis.

Bibliometric analysis is a scientometric method that can analyze publications quantitatively [13–17]. In bibliometric analysis, citation analysis is the most common method, furthermore, the results can be presented as a visualized map. Articles with the most citations analyzed bibliometrically in one field can reveal the most important contributors and the research hotspots using a quantitative method [18]. To our knowledge, no bibliometric analysis of TAO research has been performed. In this study, TAO's top 100 cited articles are reviewed and their characteristics are analyzed.

## Methods

### Literature search

A retrieval of the Web of Science (WOS) database was performed, including Core Collection (1980–present), BIOSIS Previews (2000–2014), KCI-Korean Journal Database (1980–present), Medline (1950–present), and SciELO Citation Index (2002–present). The following search strategy was used, including (Thromboangiitis Obliterans) (topic) or (Buerger's Disease) (topic) or (Buergers Disease) (topic) or (Disease, Buerger's) (topic) or (Thromboangitis Obliterans) (topic) or (Buerger Disease) (topic) or (Disease, Buerger) (topic).

### Literature screen

The search results were sorted by citations: highest first. Two authors (Liu ZX and Ning WW) screened the articles independently, the divergence was solved by the discussion. The following strategy was used: Original articles, reviews, full-length proceeding papers, and case reports were included. Letters, comments, and book chapters were excluded. The top 100 highly cited articles were selected and added to the “marked list” in the WOS database.

### Performance analysis

On the WOS website, the results were analyzed, which exported the total cited times, average cited times, and the yearly trends of publications and citations. The results were further analyzed on the WOS website, including the languages, research areas, document types, authors, affiliations, and countries/regions. The citation density was used to evaluate the yearly citations since the article was published, which was calculated using the following formula: citation density = total citations × 365/(the current date—the publication date). The citation density was present in a scatter plots map weighed by publication year, a nonlinear regression was performed to show the yearly trend of citation densities, which was presented as a trendline.

### Science mapping

A list of the top 100 articles was exported to Vosviewer software (version:1.6.18, Leiden University, the Netherlands) and Citespace software (version: 6.1.R3, Drexel University, USA) for visualized analysis of author keywords [19]. Using the bibliographic coupling analysis, countries, institutions, and authors were visualized to present the major contributors in the field as well as the corporate networks. A bibliographic coupling analysis of journals was performed to present the most often published journals. By using the co-citation analysis, the cited authors and journals were analyzed, and the total citations and total link strength were calculated.

### Network analysis

Full counting co-occurrence of author keywords was performed to investigate the most frequently used keywords. Briefly, the keywords were presented in a network visualization weighted by occurrence, the density map was made to show the hotspots visually. We also performed an overlay visualization weighing the average publication year. Citespace software was used for the burst detection of author keywords. Briefly, keywords were ranked by the specific appeared years, the burst represented keywords that frequently appeared during some periods.

## Results

### An overview of publications

**Fig. 1:**
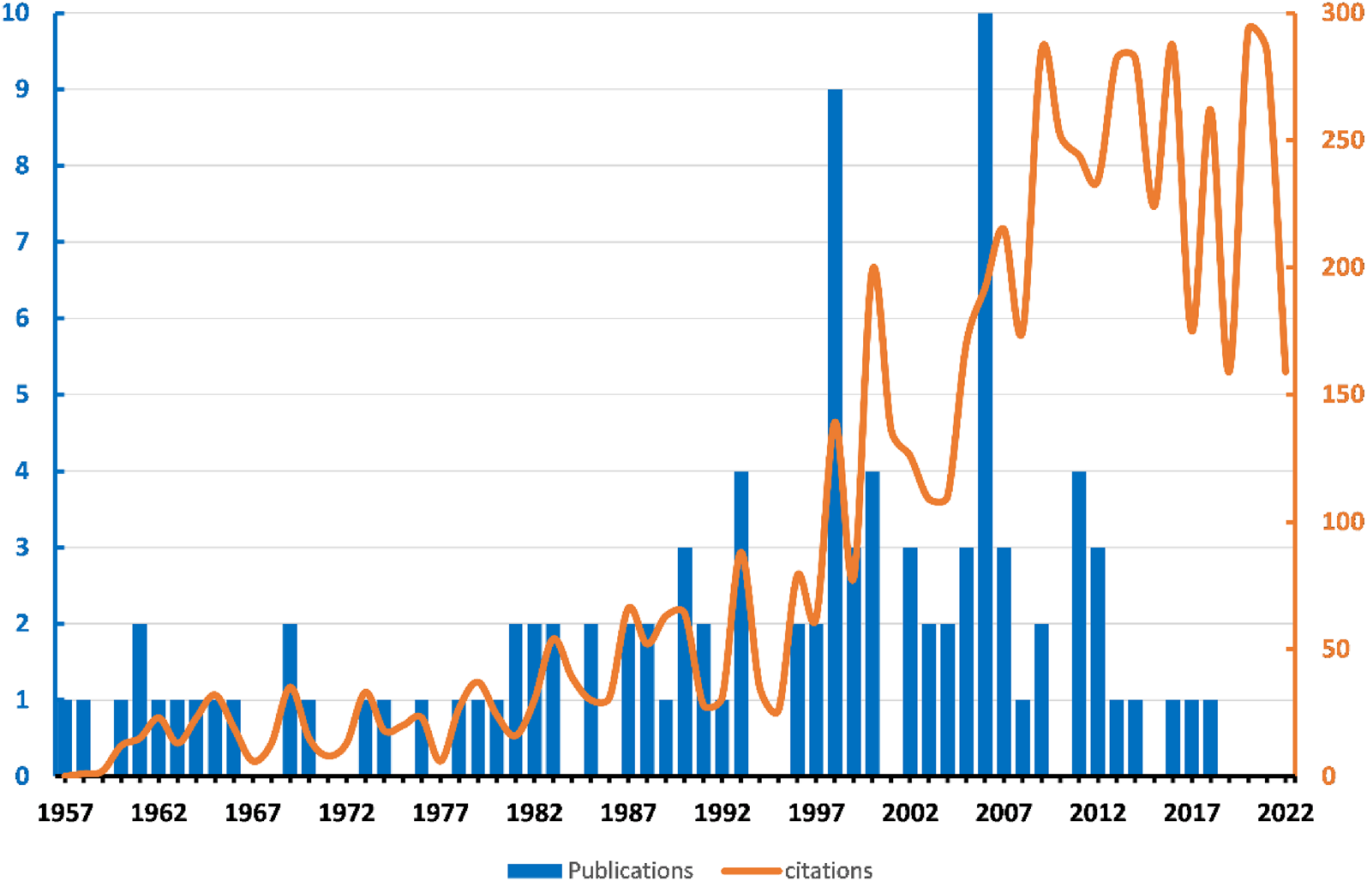
Trends of publications and citations

**Fig. 2:**
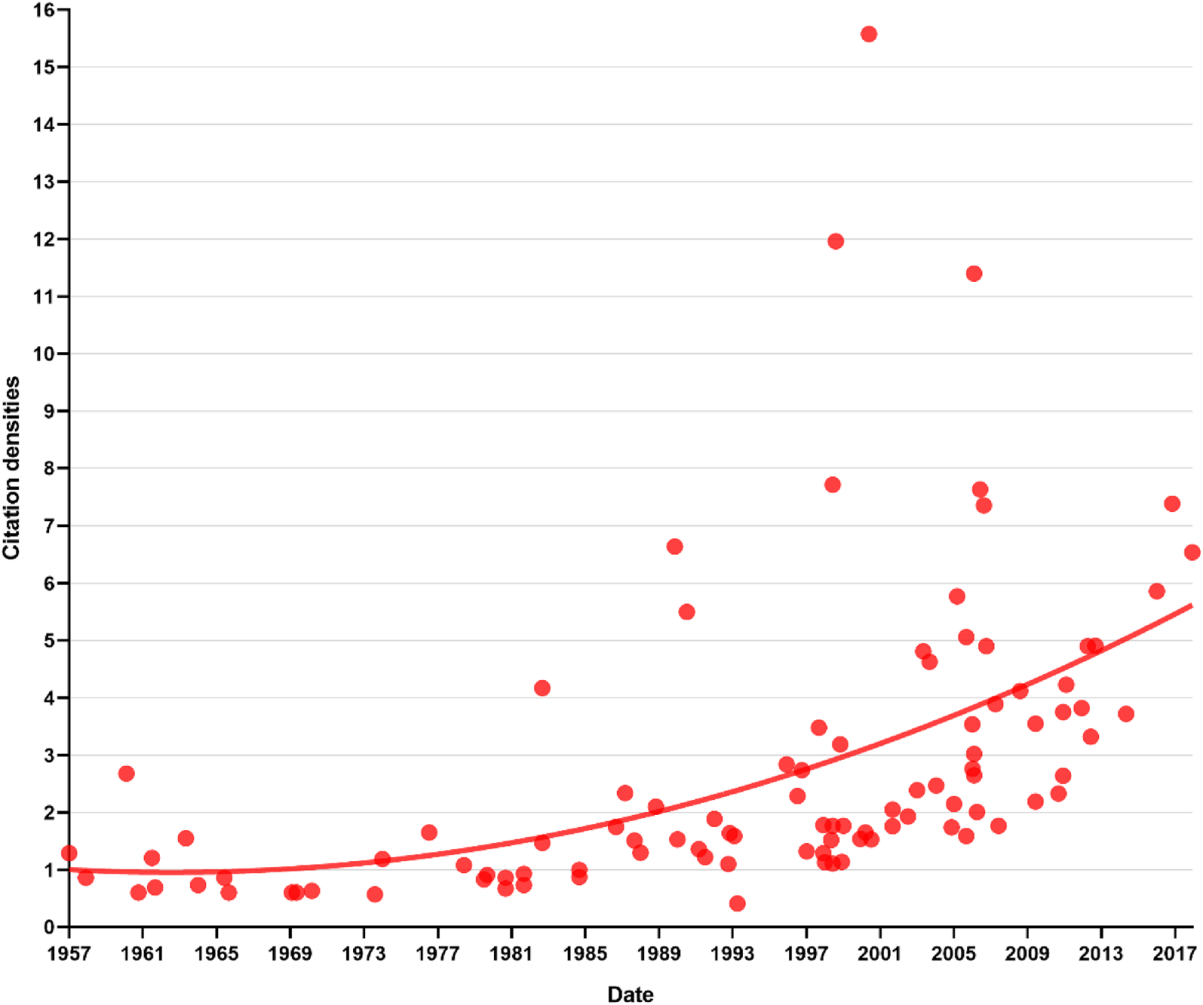
The scatter plots of citation densities are weighed by publication date, a nonlinear regression was performed

The search strategy returned 4158 results from all databases by the time we retrieved them. English was the language of all 100 of the most cited articles. There were 36 case series, 8 clinical trials, 12 case reports, 11 case–control studies, 31 reviews, and 2 experimental studies. By the time we retrieved, the top 100 cited articles had been cited 6284 times in total, and it was 5556 without self-citation. The average cited times was 62.84, and the h-index was 46. As of 1998, the yearly citations had been on a steep upward trend. The citation data for 2022 decreased because they have not been fully counted. Active years of publication were defined as more than 3 (≥ 3) top-cited articles per year. There were 11 active years of publication, among them, there were 3 highly cited articles in the year (1990, 1999, 2002, 2005, 2007, 2012), 4 highly articles in the year 1993, 2000, and 2011. The peak of highly cited articles appeared in 1998 and 2007, with 9 and 10 publications, respectively (Fig. 1). The scatter plots of citation densities showed that the majority citation density ranged from 0.5 to 3, the high citation densities intensively appeared between the years 2003 and 2015. The article with the highest citation density was “Olin, JW, Current concepts: Thromboangiitis obliterans (Buerger's disease)(2000)”, the citation density was 15.58, followed by “Isner, JM et al., Treatment of thromboangiitis obliterans (Buerger's disease) by intramuscular gene transfer of vascular endothelial growth factor: Preliminary clinical results (1998)”, the citation density was 11.96, and “Kim, Sung-Whan et al., Successful stem cell therapy using umbilical cord blood-derived multipotent stem cells for Buerger's disease and ischemic limb disease animal model (2006)”, the citation density was 11.40 (Fig. 2).

### Countries

**Fig. 3:**
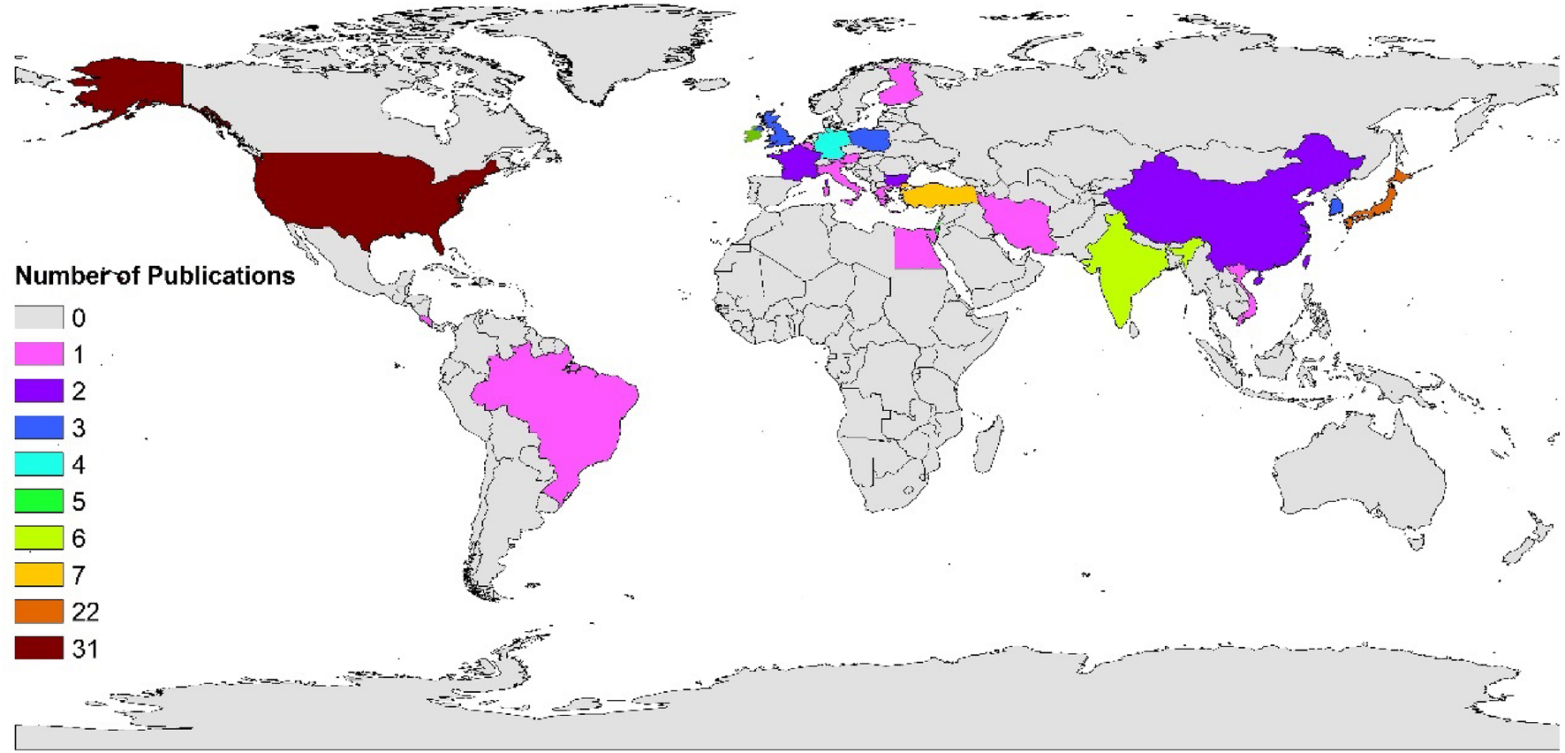
The global distribution of the top 100 cited articles

The USA and Japan contributed nearly half of the top cited articles, and they owned the overwhelming total citations, with 20 counts and 1666 cited times, 20 counts, and 1176 cited times, respectively. Germany ranked third in the number of highly cited articles. Table 1 lists the top 10 countries with the most contributions. Distribution around the world of the top 100 cited articles is shown in Fig. 3. The visualized network of a major contributor can be seen in Additional file 1: Fig. S1.

**Table 1:** Top 10 countries with the greatest number of highly cited articles

Country	Counts	Times cited	Average per item	H-index
USA	20	1666	83.3	20
Japan	20	1176	58.8	20
Germany	5	402	80.4	5
Turkey	5	272	54.4	5
Israel	4	339	84.75	4
India	4	167	41.75	4
Poland	4	156	42.25	4
South Korea	3	282	94	3
France	2	296	148	2
Bulgaria	2	74	37	2

### Institutions

Mayo Clinic and Nagoya University were the top two institutions that contributed the largest number of highly cited articles, with 6 publications and 321 cited times, 6 publications, and 302 cited times, respectively. Followed by Tokyo Medical Dental University Tmdu, Chaim Sheba Medical Center, and Sackler Faculty of Medicine. It was worth noting that Chaim Sheba Medical Center was the institution with the highest number of average citations, with 3 publications and 301 cited times. The average citation time was 100.33. Table 2 shows the top 10 institutions that contributed the most highly cited articles. In Additional file 1: Fig. S2, institutions with the highest number of highly cited articles are visualized.

**Table 2:** Top 10 institutions with the greatest number of highly cited articles

Institution	Counts	Times cited	Average citation per item	H-index
Mayo Clinic	6	321	53.5	6
Nagoya University	6	302	50.33	6
Tokyo Medical Dental University Tmdu	4	187	46.75	4
Chaim Sheba Medical Center	3	301	100.33	3
Sackler Faculty of Medicine	3	263	87.67	3
Seoul National University Snu	3	282	94	3
Tel Aviv University	3	263	87.87	3
Aichi Medical University	2	119	59.5	2
Ankara University	2	158	79	2
Asahikawa Medical College	2	102	51	2

### Authors

There were 16 sole-authored publications, among them, Lie, J. T., Shionoya, S. owned 3 sole-author publications, respectively. Wessler, S. owned 2 sole-author publications. The rest sole-authors each owned 1 article. There were 84 co-authored publications.

**Fig. 4:**
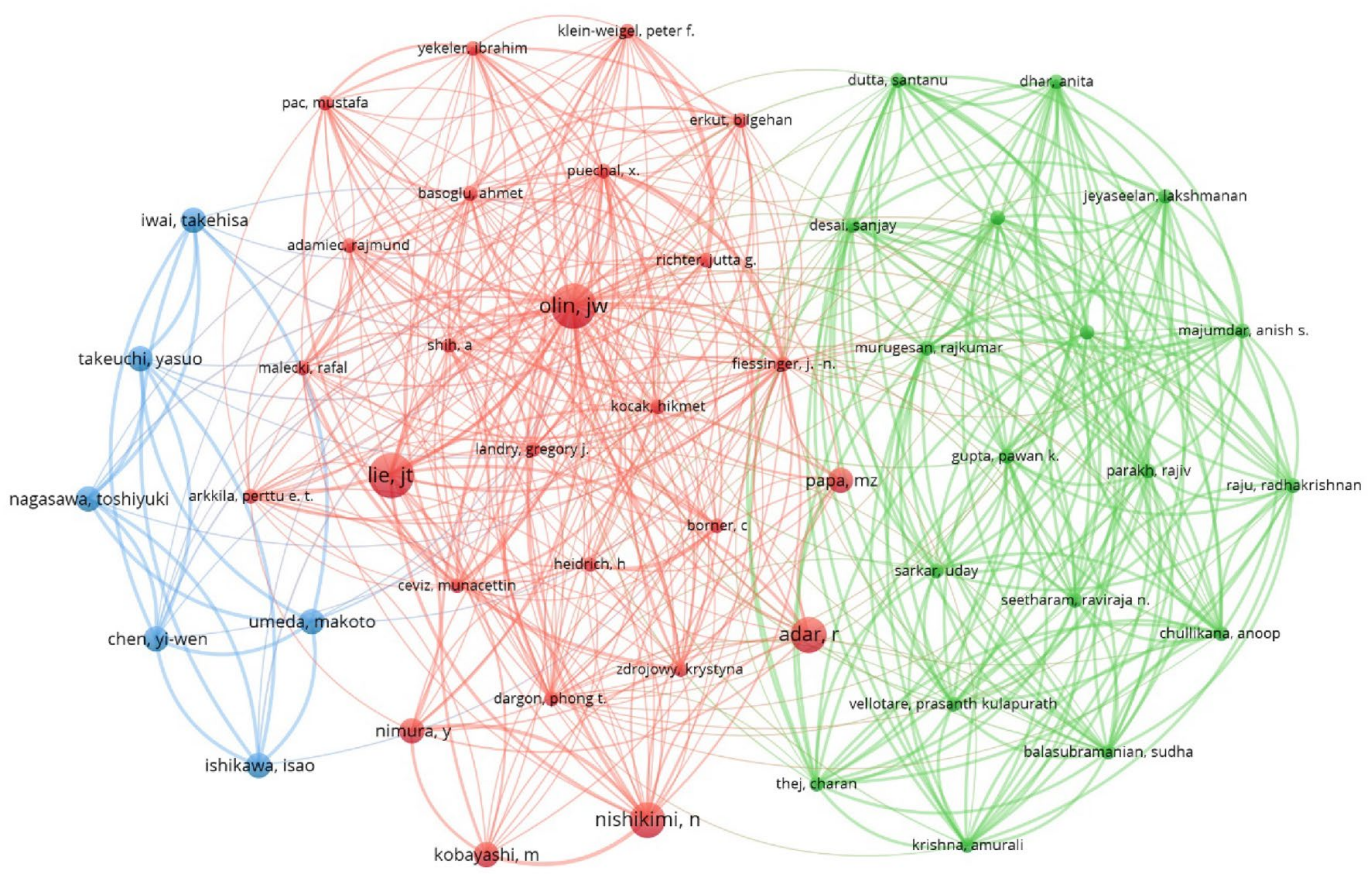
Bibliographic coupling analysis of authors

The author with the most highly cited articles was Shionoya S, with 5 articles and 394 citations, There were 78.8 citations per item on average. The author with the most average citations per item was Olin JW, with 4 articles and 652 citations, There were 163 citations per item on average, followed by Iwai T, and Lie JT. Table 3 shows the top 10 authors with the highest number of highly cited articles. In Fig. 4, the authors with the greatest number of highly cited articles are visualized.

**Table 3:** Top 10 authors with the greatest number of highly cited articles

Author	Affiliation^a^	Counts	Times cited	Average citation per item	H-index
Shionoya S	SL Medical Group	5	394	78.8	5
Olin JW	Mt Sinai Sch Med	4	652	163	4
Iwai T	Tokyo Med & Dent Univ	4	188	47	4
Lie JT	Mayo Clin & Mayo Fdn	4	232	58	4
Adar R	Chaim Sheba Med Ctr	3	301	100.33	3
Wessler, S	Beth Israel Hospital	3	237	79	3
Ishikawa, I	Tokyo Med & Dent Univ	3	157	52.33	3
Umeda M	Tokyo Med & Dent Univ	3	157	52.33	3
Nishikimi, N	Nagoya Univ	3	140	46.67	3
Chen, YW	Tokyo Med & Dent Univ	2	56	28	2

^a^Institution marked in the article, does not represent the present affiliation

The author with the most references was Shionoya S, who was cited 90 times, and there were 748 link strengths. It is Olin JW who is the second most frequently cited author in the references. He was cited 73 times, and the link strength was 551. It was followed by Lie JT, who had 72 citations and totaled 726 link strength. Table 4 lists the top 10 highly cited authors in the references. Additional file 1: Fig. S3 shows the co-citation analysis of the highly cited authors in the references.

**Table 4:** Top 10 cited authors in the references

Author	Affiliation^a^	Citations	Total link strength
Shionoya S	SL Medical Group	90	748
Olin JW	Mt Sinai Sch Med	73	551
Lie JT	Mayo Clin & Mayo Fdn	72	726
Buerger L	Mt. Sinai Hospital	54	548
Mills JL	University of South Florida College of Medicine	39	365
Adar R	Chaim Sheba Med Ctr	37	324
Mckusick, VA	Johns Hopkins Univ	33	368
Wessler, S	Beth Israel Hospital	29	268
Fiessinger, JN	European Georges Pompidou Hosp	23	226
Sasajima, T	Asahikawa Med Univ	21	186

^a^Institution marked in the article, does not represent the present affiliation

### Journals

With 9 articles and 858 citations, the Journal of Vascular Surgery had the most highly cited articles. With 5 articles and 434 citations, Circulation ranked second, while the International Journal of Cardiology was third with 5 articles. They were followed by the European Journal of Vascular and Endovascular Surgery (4 articles), Annals of Vascular Surgery (4 articles), Surgery (3 articles), American Journal of Surgery (2 articles), Atherosclerosis (2 articles), Archives of Internal Medicine (2 articles), and Angiology (2 articles) (Table 5). Additional file 1: Fig. S4 shows a map of the journals where the most highly cited articles were published.

**Table 5:** Top 10 most often published journals

Journal	Times counted	Citations	Average citation per item	Impact factor (2021)
Journal of Vascular Surgery	9	858	95.33	4.860
Circulation	5	434	86.8	39.918
International Journal of Cardiology	5	317	63.4	4.039
European Journal of Vascular and Endovascular Surgery	4	238	59.5	6.427
Annals of Vascular Surgery	4	157	39.25	1.607
Surgery	3	153	51	4.348
American Journal of Surgery	2	110	55	3.125
Atherosclerosis	2	91	45.5	6.847
Archives of Internal Medicine	2	70	35	NA
Angiology	2	66	33	3.299

There were most references to Circulation in the references, a total of 1080 link strengths 116 times were cited for this article. In the references, Angiology was the second most frequently cited journal, a total of 1080 link strengths 103 times were cited for this article. The third was the Journal of Vascular Surgery, a total of 1283 link strengths 99 times were cited for this article. Additional file 1: Table S1 lists the top 10 most cited journals in the references. The top 100 articles are listed in Additional file 1: Table S2. In Additional file 1: Fig. S5, we showed the co-citation analysis of the highly cited journals in the references.

### Research area

**Fig. 5:**
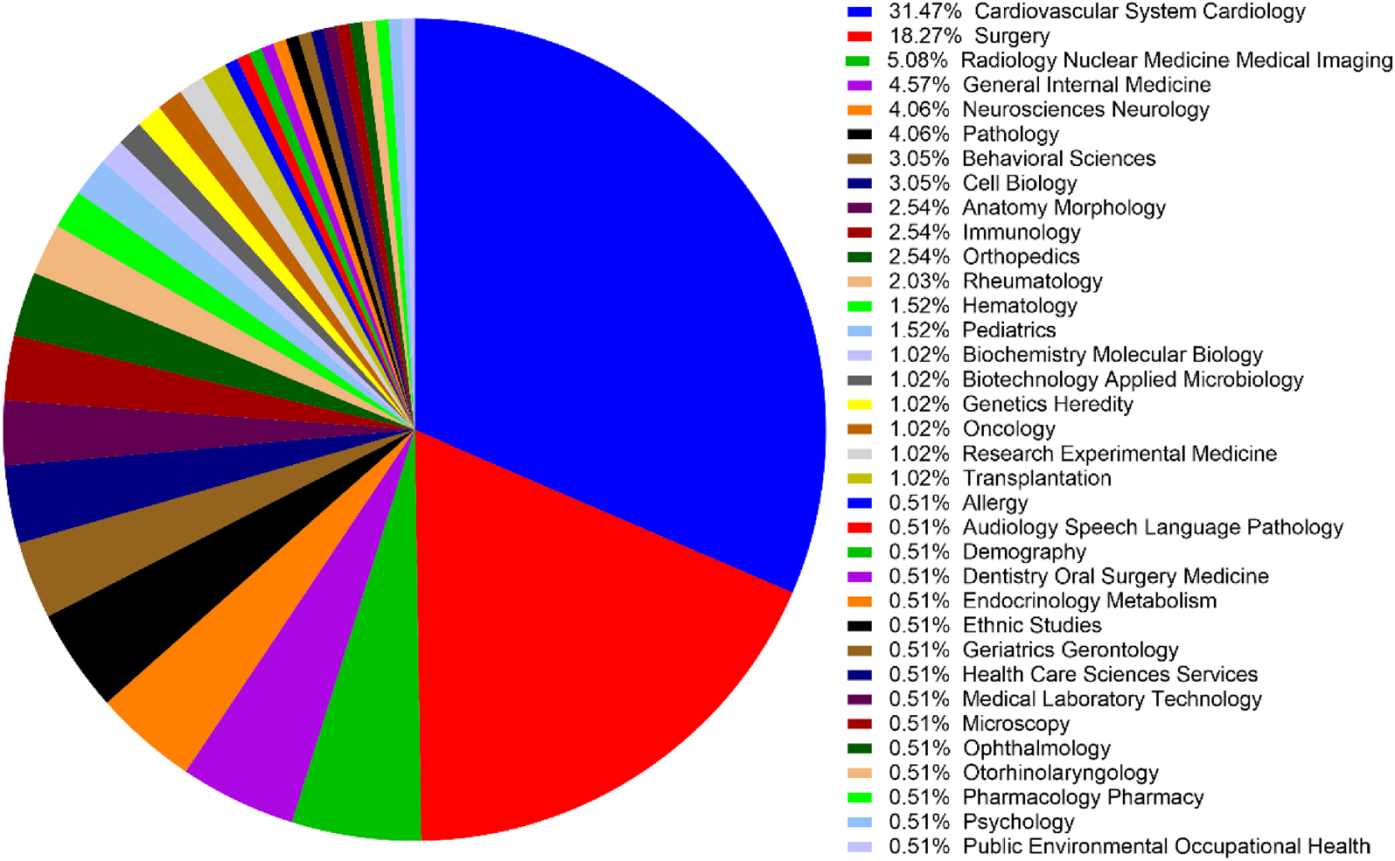
The distribution of the research areas in the top 100 cited articles

The top 10 most focused research areas were Cardiovascular System Cardiology, Surgery, Radiology Nuclear Medicine Medical Imaging, General Internal Medicine, Neurosciences Neurology, Pathology, Behavioral Sciences, Cell Biology, Anatomy Morphology, and Immunology. Figure 5 shows the full research area of the top 100 cited articles.

### Hotspots

**Fig. 6:**
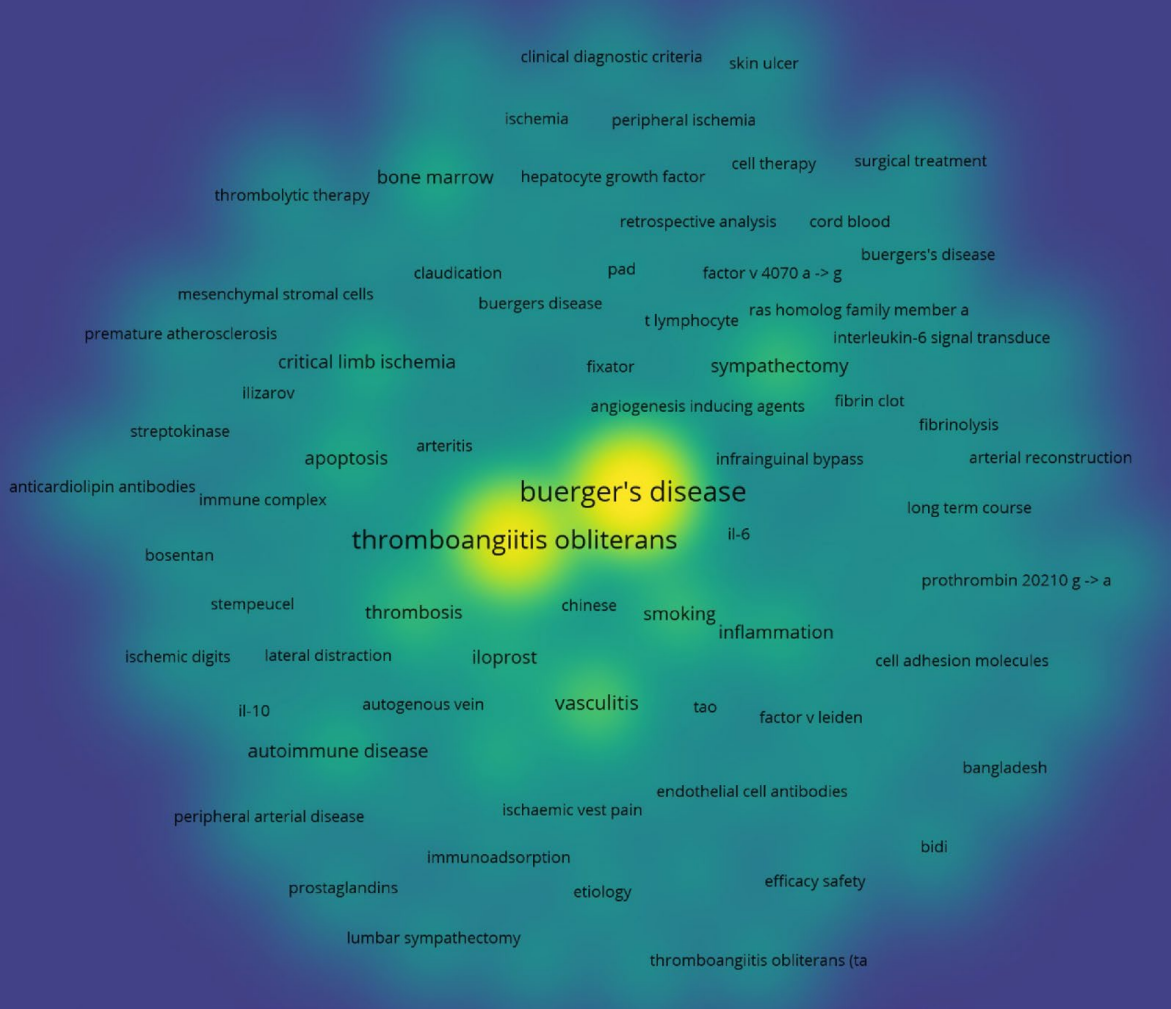
Co-occurrence analysis of author keywords, density map. *Note*: In the Vosviewer software, all the capital words were canceled and TAO was presented by lower case letters “tao”

**Fig. 7:**
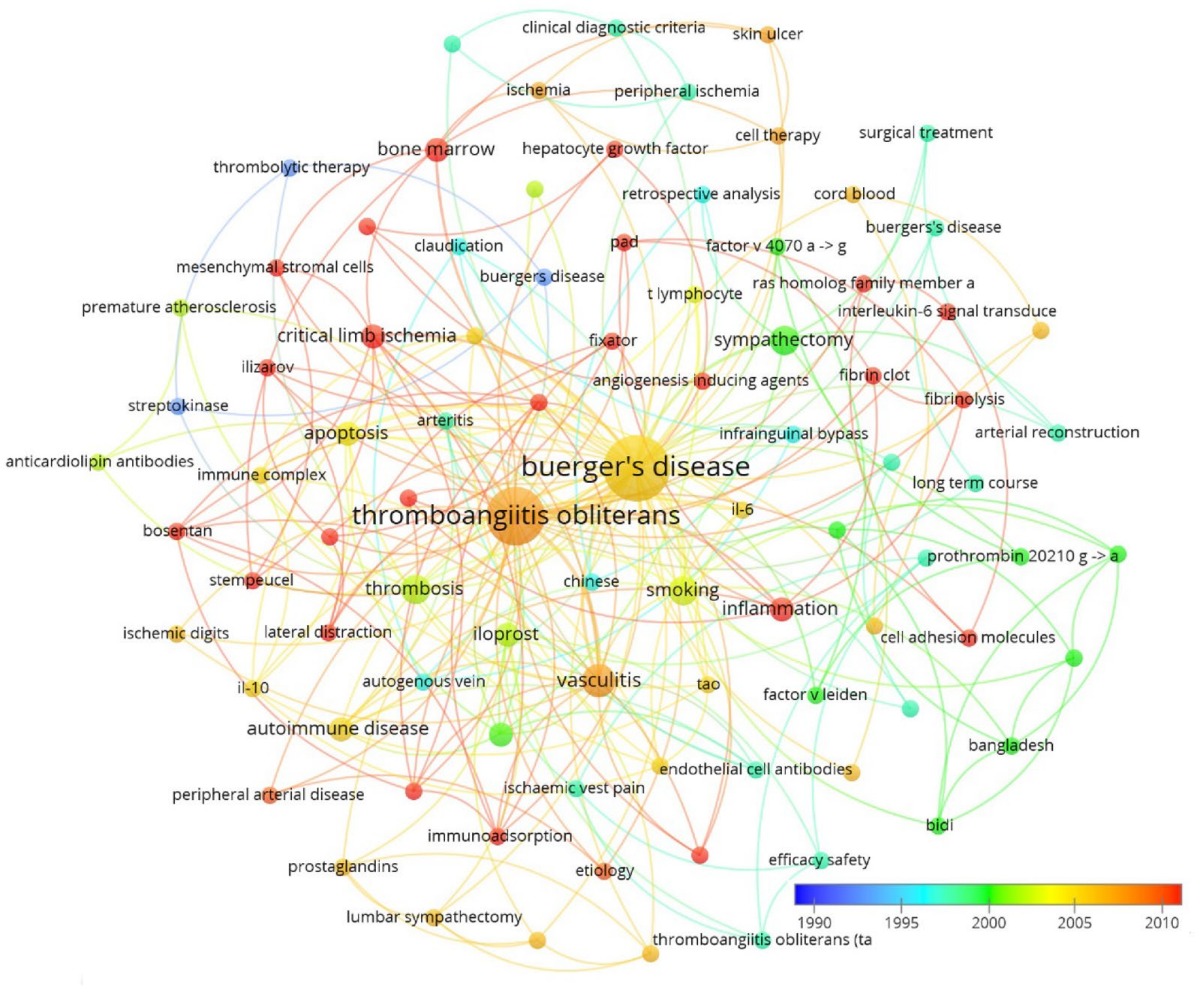
Bibliometric analysis of author keywords, ranging by year

**Fig. 8:**
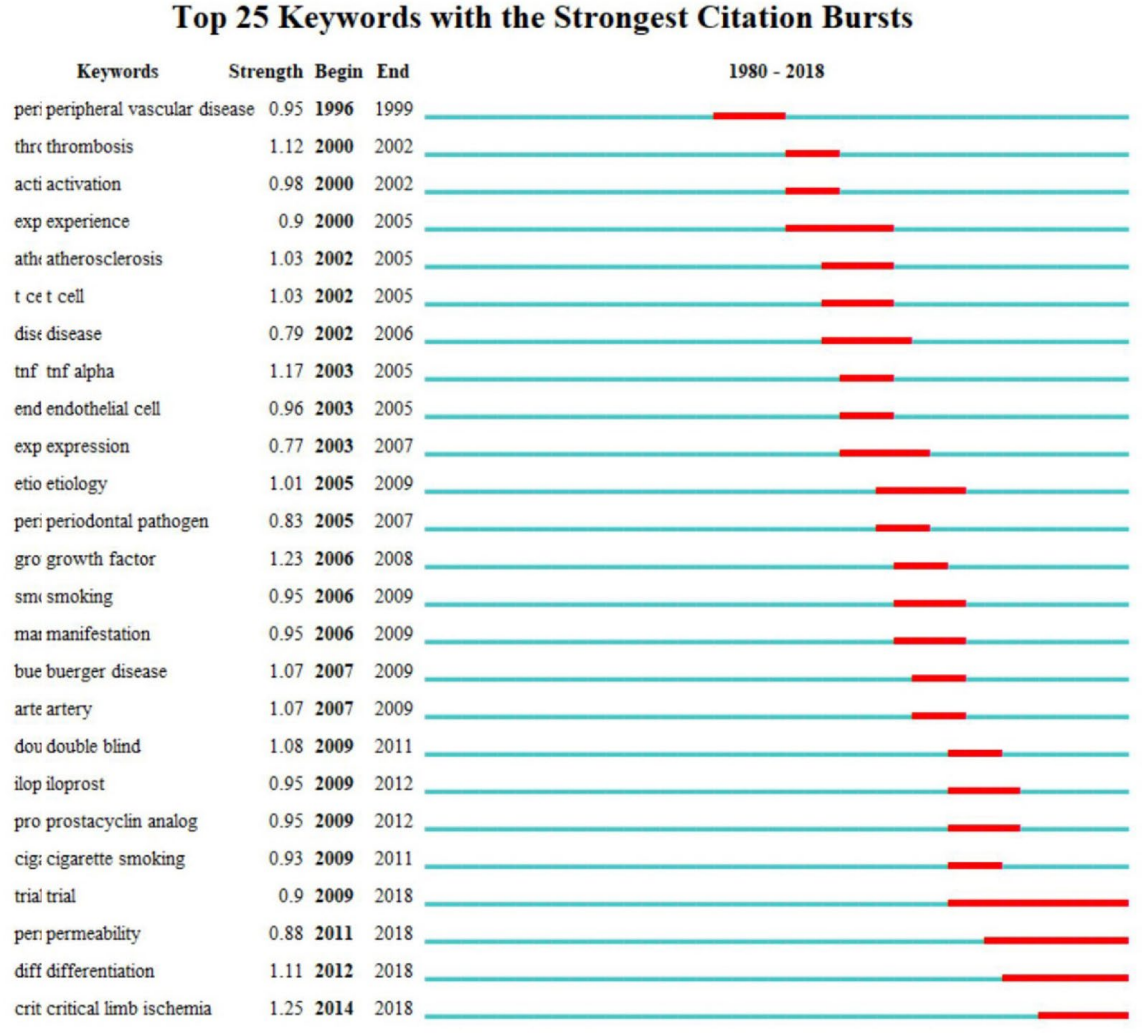
Burst detection of top 25 author keywords

The top 10 occurred keywords were “Burger’s disease”, “thromboangiitis obliterans”, “vasculitis”, “smoking”, “thrombosis”, “sympathectomy”, “autoimmune disease”, “critical limb ischemia”, “endothelial cells”, and “inflammation” (Fig. 6). When ranked by average appeared years, Recently, the following keywords were frequently used: “critical limb ischemia”, “stem cell therapy”, “progenitor therapy”, “immunoadsorption”, and “mesenchymal stromal cells”, “bone marrow”, and “inflammation” (Fig. 7). The burst detection of author keywords revealed the following: “trial”, “permeability”, “differentiation”, and “critical limb ischemia” are keywords that have been burst in recent years (Fig. 8).

## Discussion

In this study, we analyzed the characteristics of the top 100 cited articles in the field of TAO. The literature review confirmed that this is the first bibliometric analysis of Burger's disease or TAO.

Bibliometric analysis has been widely used in the assessment of scientific works and is a necessary index for the evaluation of performance in research institutions, schools, and funding providers [20–23]. Citation analysis is the most subjective and important index in the evaluation of the scientific value of published literature [24–26]. For example, the journal citation reports in the WOS database calculate and analyzes the subjective citation data for the journals, export impact factors, and evaluate the authority and influence of publishers. Though the scientific impact can not be fully reflected by the impact factors, “highly cited articles” do not fully represent “the most influential articles”. Furthermore, the inequality of global citation is also a new tendency in citation analysis [27]. The citation density is a tool that was widely used in the citation analysis, which can avoid the bias that was caused by the “age of literature”. We noticed that articles published after 2003 have higher citation densities, inflecting their high academic values. However, the impact of newly published articles can be underestimated, which is an inevitable problem in citation analysis. Despite these facts, this study well presented several visualized maps of the TAO's top 100 cited articles, The major contributors and hotspots were identified, and we believe the results are useful for the researchers.

The calculation of citation density was employed as a measure to offset the potential "age advantage" inherent in citation counts. This is crucial because the total citations of recently published articles can be erroneously underestimated due to the age of the literature. In other words, the annual citation rates can be influenced by the age of the publications. Surprisingly, our analysis revealed a notable upward trajectory in citation densities. Contrary to expectations, the most recent publications exhibited higher citation densities when compared to older articles. It is essential to acknowledge that the density data could be impacted by the significant surge in citations since 1997, as reflected in Fig. 1, which accounts for the increased overall citation counts. In summary, our findings suggest that publication date does play a partial role in citation density. Recent articles tend to receive more frequent citations, even in the face of a substantial upsurge in publications in recent years. Why do these recently published articles garner greater citation rates? We hypothesize that they likely delve into the most intriguing areas and offer fresh insights into the field of TAO research.

Within the top 100 most highly cited articles, 31 of them were review papers. The majority of these reviews provided comprehensive insights into the concepts of TAO from a holistic perspective. They covered current concepts (the most cited review, with 347 citations as we analyzed), diagnosis (ranked fifth in citation count), critical evaluation (seventh in citations), as well as comprehensive summaries of diagnosis, features, and therapy (ranking twelfth, thirty-third, thirty-seventh, and forty-second). Notably, there was an article titled "Thromboangiitis obliterans/Buerger's disease" that ranked sixteenth, twenty-ninth, thirtieth, thirty-fifth, thirty-sixth, and thirty-eighth in citations, indicating that reviews that extensively explored TAO's concepts and characteristics tended to attract more citations.

Among these top 100 articles, 56 were original research articles. The second and third most highly cited articles were clinical trials that focused on the treatment of TAO through intramuscular gene transfer of vascular endothelial growth factors and the effects of iloprost on the treatment of limb ischemia in TAO. Furthermore, there were eight clinical trials within the top 100 highly cited articles, three of which investigated the clinical effects of different bone marrow stem cells in treating TAO, alongside three case series. Both case series and review articles accounted for one-third of the top 100 highly cited articles. Case series frequently explored research areas such as cellular infiltration, inflammation, autoimmunity, and immunohistochemical or immunobiologic analysis. In essence, these highly cited articles often concentrated on the immunological mechanisms and surgical treatments of TAO. Within the 11 case–control studies, four measured the levels of autoantibodies in TAO, including antiphospholipid antibodies, antiendothelial cell antibodies, and cytokine production, reflecting the focal points in the fundamental mechanisms of TAO research. The choice of study design highlighted the prevalence of case series studies in the clinical study of TAO, as case–control studies and clinical trials tend to be more challenging to implement compared to retrospective case series. Interestingly, we observed that the levels of evidence did not correlate with the total citation count. Among the 12 case reports, aside from summarizing early-stage recognition of the disease, most of them focused on rare manifestations or the mortality of TAO. Two case reports delved into the pathogenesis of TAO. Additionally, two experimental studies explored the efficiency of stem cell therapy in animal models and the expression of cell molecules and cytokines in the endothelium of arteries in TAO.

The reason these papers garner higher citations than others lies in their ability to encapsulate the prevailing hotspots and contemporary trends within the realm of TAO research. These aspects held particular favor within a specific period of TAO research.

The present study helps researchers locate the most influential authors, institutions, and countries by revealing the most highly cited authors, institutions, and countries, as well as gathering the research area of TAO. This study found that JAPAN and USA contributed overwhelming numbers of highly cited articles, suggesting their authority in this field. Though the epidemiology of TAO concluded that the prevalence of TAO decreased in JAPAN [3], top-cited TAO research contributed more from Japan than from other countries. In terms of highly cited institutions, Mayo Clinic and Nagoya University owned the largest number of highly cited articles. Articles from the Chaim Sheba Medical Center and Sackler Faculty of Medicine were highly cited per item.

The highly cited authors were revealed both by analyzing the total counts in the WOS online and by identifying highly cited authors in the references based on their co-citations. Notably, Shionoya S, Lie JT, Olin JW, and Buerger L were authors that both owned the largest number of highly cited articles and highly cited authors in the references, suggesting their authority and original contributions to the TAO research.

In the field of vascular research, articles with the highest citations tended to be published in specialized journals, and the impact factors were not comparable to the total cited times, which further demonstrated that the academic value of publications cannot be fully represented by the impact factor. In the references, we conducted a co-citation analysis of highly cited journals, which revealed similar results. It has been reported that the current evaluation system paid excessive focus on impact factors, for the impact factors were hugely influenced by the yearly publications and improper citations, the academic value of literature or the influence of the publisher should be evaluated comprehensively [28]. More feasible use of impact factors in the bibliometric analysis has been appealed to by researchers [29–31].

By analyzing the co-occurrence of author keywords and the research area, hotspots were identified, as well as burst detection of author keywords. It was not surprising that the majority of the highly cited articles focused on the cardiovascular system. TAO is a vasculitis that affects the small and medium-sized arteries, though the ischemia of the limb is the main manifestation [6, 8, 32]. The second most focused research area was surgery, suggesting that the highly cited articles more often investigated the surgical intervention for the TAO, and the management of critical limb ischemia is the hotspot of TAO research in recent years, which was revealed by the average appeared year of keywords.

We found that the hotspots of bibliometric analysis are compatible with the existing challenges of TAO research, the top cited articles highly concentrated on the trends and apparent questions and challenges of TAO, along with the different research stages and understanding of the disease. The reviews (counted one-third in the top 100 cited articles) summarized the pathology, diagnosis, evaluation, therapy, and new progress of the disease, except for smoking, and exposure to cold environments, the current studies consistently support the view that TAO is an autoimmune disease, the antiphospholipid antibodies, collagen antibodies, complements, and autoimmune cytokines were found in the blood of TAO patients. The places that researchers are looking at represent the hotspots in a certain period, however, it cannot always reflect the right places to look, for example, the illustration of pathology and surgical treatment of TAO. Thromboangiitis obliterans is now generally accepted as an autoimmune disease; besides, clinical findings revealed that thromboangiitis obliterans patients tend to familial aggregation and genetic predisposition [8]. The novel findings in the mechanism of diseases incubated the auto-immunological therapies, especially autologous cell therapy [4]. The knowledge gaps and novel ideas for investigation can be revealed by the analysis of hotspots of these top-cited articles.

We constructed two diagrams to visually represent the research domains of original articles and reviews. These diagrams were created based on their categorizations, which encompassed epidemiology, etiology, pathophysiology, diagnosis, treatment, and prognosis (Additional file 1: Tables S3 and S4). In the case of pathophysiology, we further segmented it into distinct sections, delineating various aspects such as the immune system, genetics, endothelial dysfunction, autoimmunity, inflammation, coagulation and thrombosis, genetics, and epigenetics. Likewise, the treatment category underwent a refined classification, differentiating between medicine, endovascular surgery, cellular therapy, and innovative therapeutic approaches.

Within the realm of original articles, encompassing clinical trials, case series, and case–control studies, our investigation revealed that treatment and pathophysiology were the most frequently addressed aspects. Interestingly, manifestations, etiology, diagnosis, prognosis, and epidemiology garnered relatively less attention. This suggests that original research predominantly centered around the treatment of TAO, often sidelining the assessment of prognosis. In the case of review articles, a different pattern emerged, with an emphasis on summarizing treatment and diagnosis, closely followed by discussions on pathogenesis. In contrast, manifestation, prognosis, and epidemiology received comparatively less coverage. In summation, reviews predominantly concentrated on diagnosis, and treatment was a recurring focal point in the top 100 cited articles. The relative lack of interest in epidemiology can be attributed to the uneven distribution of TAO globally, particularly its higher prevalence in certain regions with distinct characteristics, like high mortality in the East. The manifestation of TAO presented clearer patterns, marked by the identification of typical symptoms. It is notable that prognosis featured less prominently in therapeutic research, often being overlooked. In terms of pathophysiology, the immune system, notably autoimmunity, emerged as a prominent area of interest. Inflammation and infectious factors constituted the most captivating subjects within the highly cited articles, aligning with the hotspots uncovered by network and burst keyword analysis.

In detail, the most recent highly cited TAO research (since 2015) mostly focused on stem cell therapy, progenitor therapy, immunoadsorption, mesenchymal stromal cells, bone marrow, and inflammation, which represents the newest promising treatment strategy for TAO [33–37]. Burst detection revealed keywords that frequently appeared in some consecutive periods, the results showed that critical limb ischemia burst from 2014 to 2018, which was consistent with the results that were exported from Vosviewer. It must be emphasized that the co-occurrence analysis counts the average year keywords appear, while burst detection counts the specific year keywords appear. Thus, the consistency of the results is inevitable.

This study aimed to unveil the distinctive characteristics of the top 100 most cited papers in the field of TAO research, with the hope that it will prove valuable for researchers with an interest in TAO. TAO exhibits differing prevalence across regions, with a higher occurrence in the Middle East, Siberia, Japan, South Korea, and certain areas, while remaining relatively rare in America, Western, and Northern Europe [3–5]. Interestingly, we observed that Japan held the second-highest number of highly cited articles among the top 100, closely followed by Turkey, India, Israel, Germany, and South Korea. These findings align with the geographic distribution of TAO cases. Moreover, it is worth noting that the United States secured the top rank both in citation counts and the number of related articles, possibly reflecting its significant global academic influence. Nonetheless, it is essential to acknowledge the limitations of this analysis, as it only considered the top 100 highly cited articles in the field of TAO, thereby offering only a partial reflection of TAO's epidemiology.

This study has some limitations. First, it is a bibliometric analysis based on the WOS database, other databases were not retrieved, and the representative of the search results is limited. Second, the distribution of institutions and countries was mostly dependent on the authors; however, the counts of the contribution in the WOS database were based on the appeared times, which may lead to bias. Third, hotspots extracted from the 100 articles cannot fully represent the real research interest in this field. Fourth, the value of citation analysis is limited when it comes to the evaluation of academic impact, which should be evaluated comprehensively. Fifth, within the VOSviewer software, it was observed that all capitalized words were rendered in lowercase letters, including "TAO", and this also applied to other keywords. Unfortunately, this formatting limitation hinders the accurate representation of the primary topic.

## Conclusions

This study revealed the highly cited contributors of research on TAO. Most cited articles in the top 100 focused on the cardiovascular system and surgery. Treatment and pathophysiology including stem cell therapy, progenitor therapy, genetics, autoimmunity, and inflammation are the hotspots of TAO.

### Supplementary Information

**Additional file 1. Fig. S1**: Bibliographic coupling analysis of countries. **Fig. S2**: Bibliographic coupling analysis of institutions. **Fig. S3**: Co-citation analysis of highly cited authors in the references. **Fig. S4**: Bibliographic coupling analysis of journals. **Fig. S5**: Co-citation analysis of highly cited journals in the references. **Table S1**: Top 10 most cited journals in the references. **Table S2**: List of the top 100 cited articles. **Table S3**: Visual representation of the research domains of original articles. **Table S4**: Visual representation of the research domains of reviews.

## Data Availability

All data generated or analyzed during this study are included in this published article and its Additional file 1.
