# Supplementary material for: Top 100 cited articles in the thromboangiitis obliterans: a bibliometric analysis and visualized study
Source: Eur J Med Res. 2023 Dec 2;28:551. doi: 10.1186/s40001-023-01540-6 (PMC10693135; doi:10.1186/s40001-023-01540-6)
Supplement: Supplementary file 1 — Additional file 1. Fig. S1: Bibliographic coupling analysis of countries. Fig. S2: Bibliographic coupling analysis of institutions. Fig. S3: Co-citation analysis of highly cited authors in the references. Fig. S4: Bibliographic coupling analysis of journals. Fig. S5: Co-citation analysis of highly cited journals in the references. Table S1: Top 10 most cited journals in the references. Table S2: List of the top 100 cited articles. Table S3: Visual representation of the research domains of original articles. Table S4: Visual representation of the research domains of reviews. [file 40001_2023_1540_MOESM1_ESM.docx]

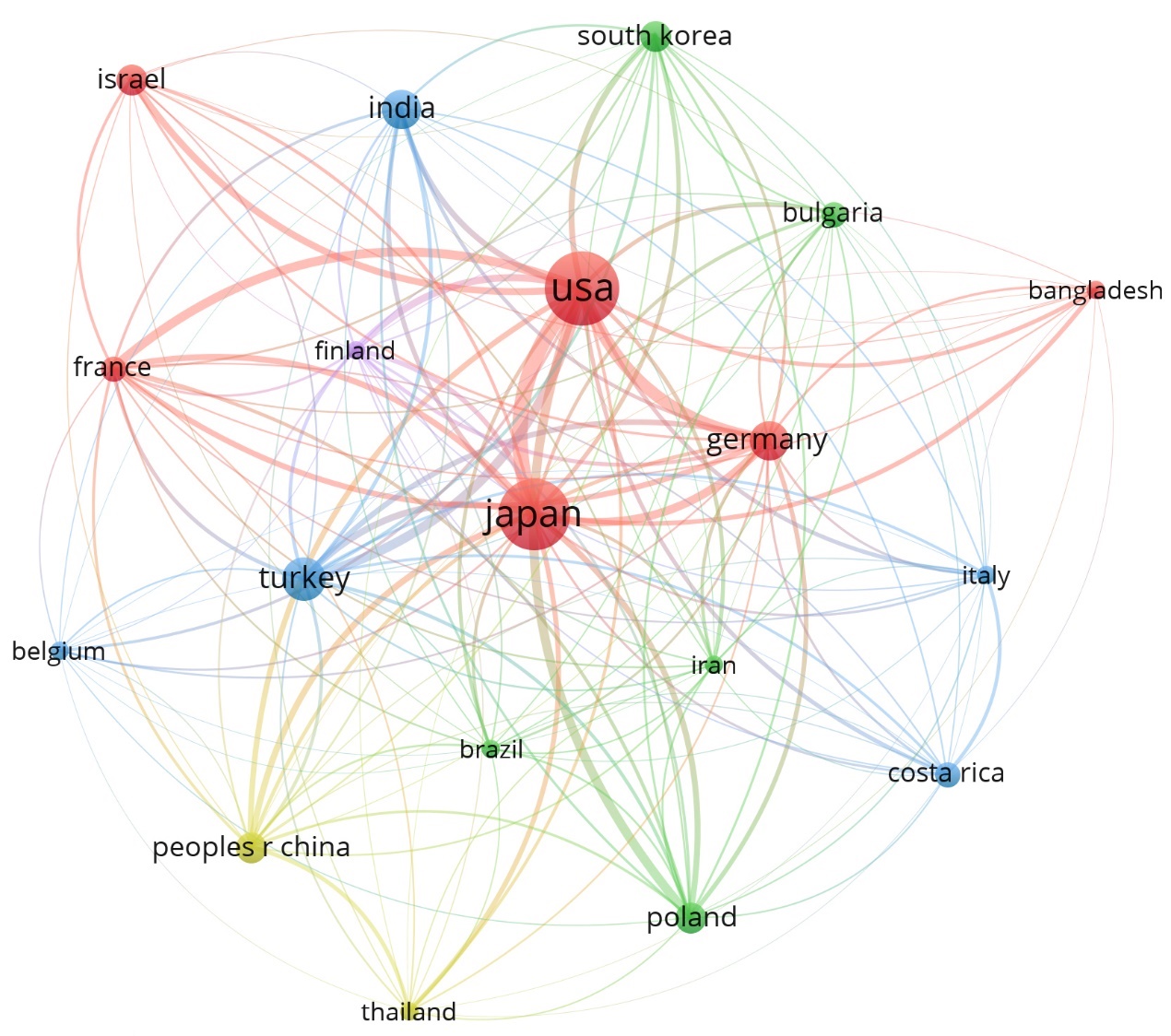
**Supplementary Fig. 1**  Bibliographic coupling analysis of countries


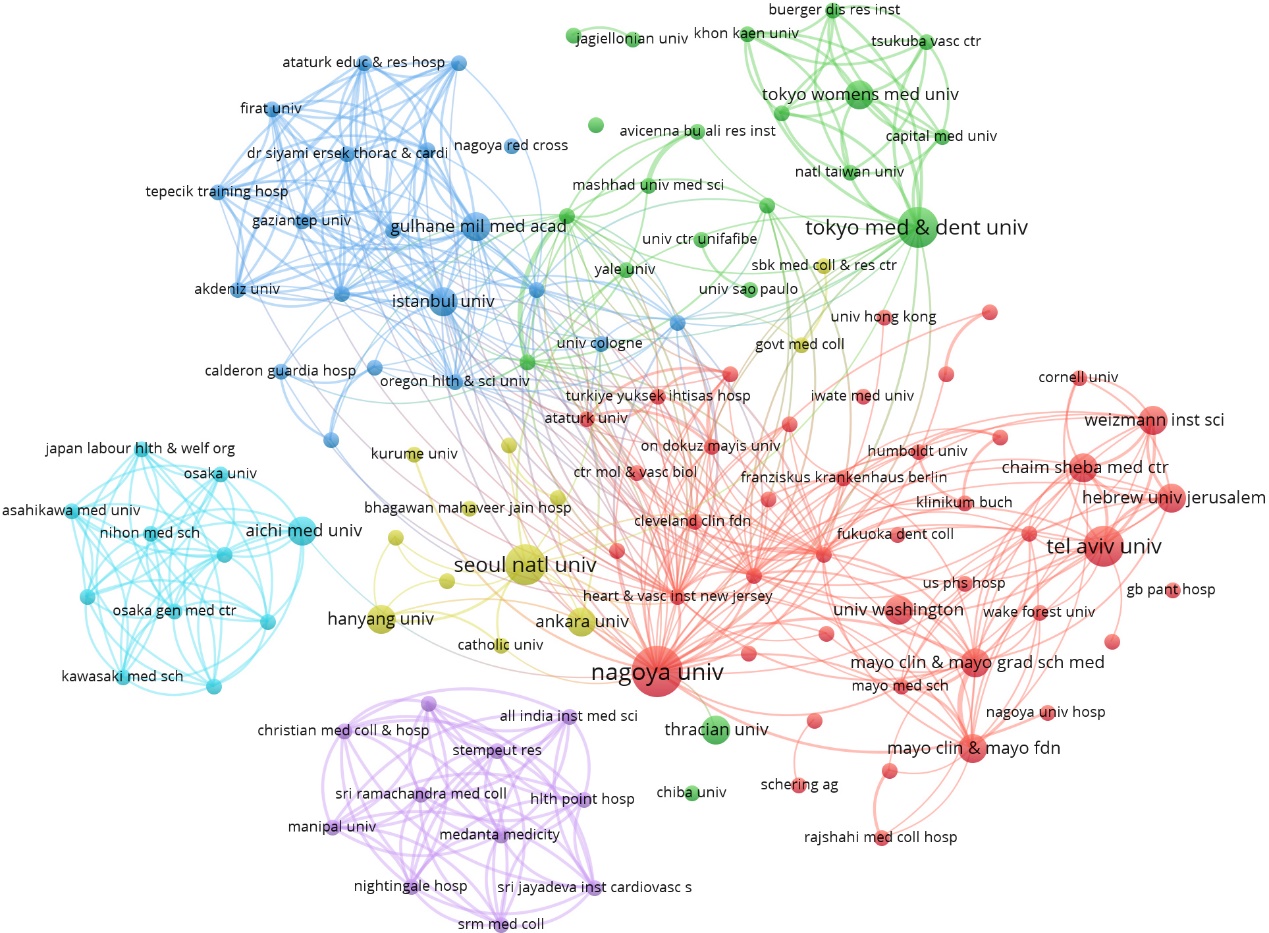
**Supplementary Fig. 2**  Bibliographic coupling analysis of institutions


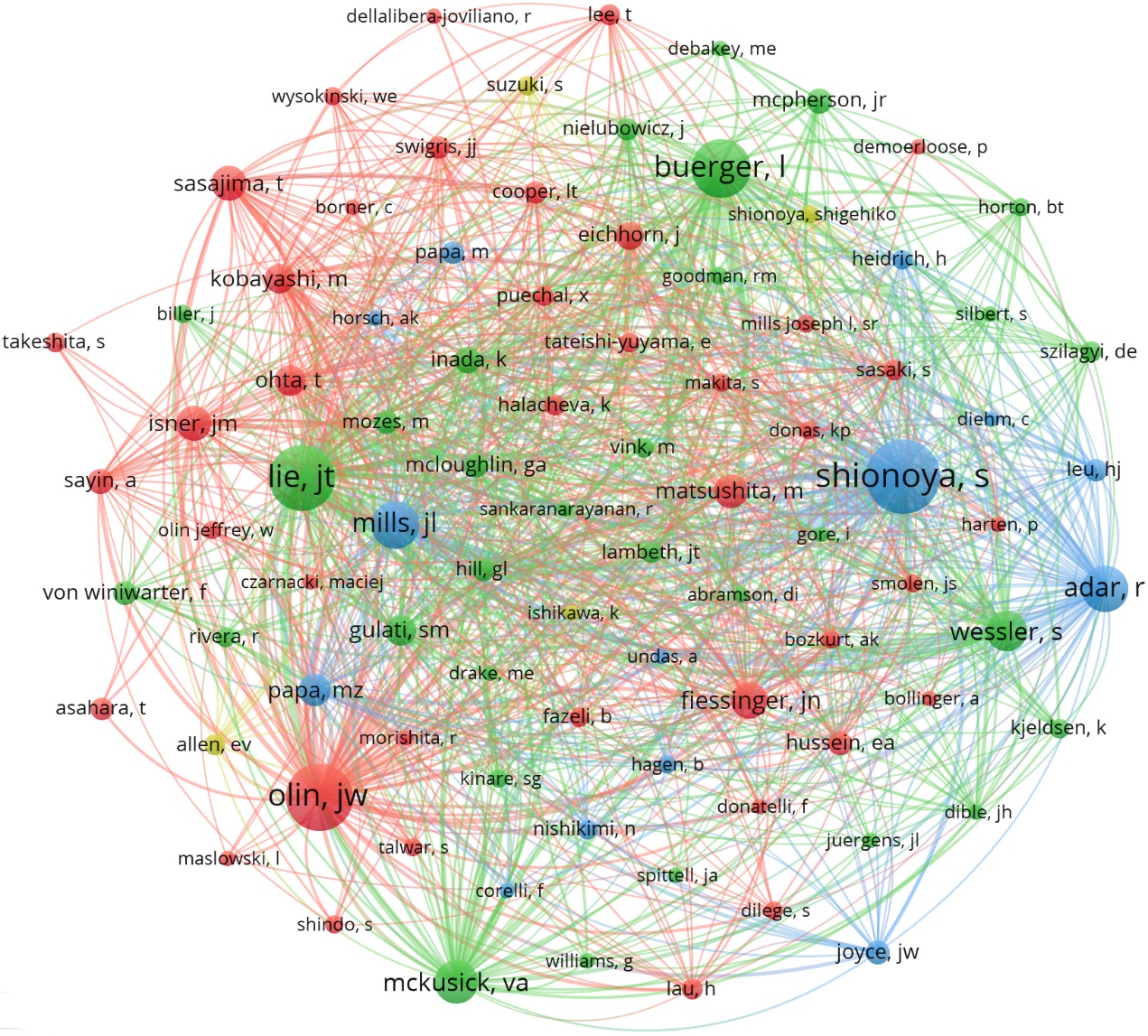
**Supplementary Fig. 3**  Co-citation analysis of highly cited authors in the references


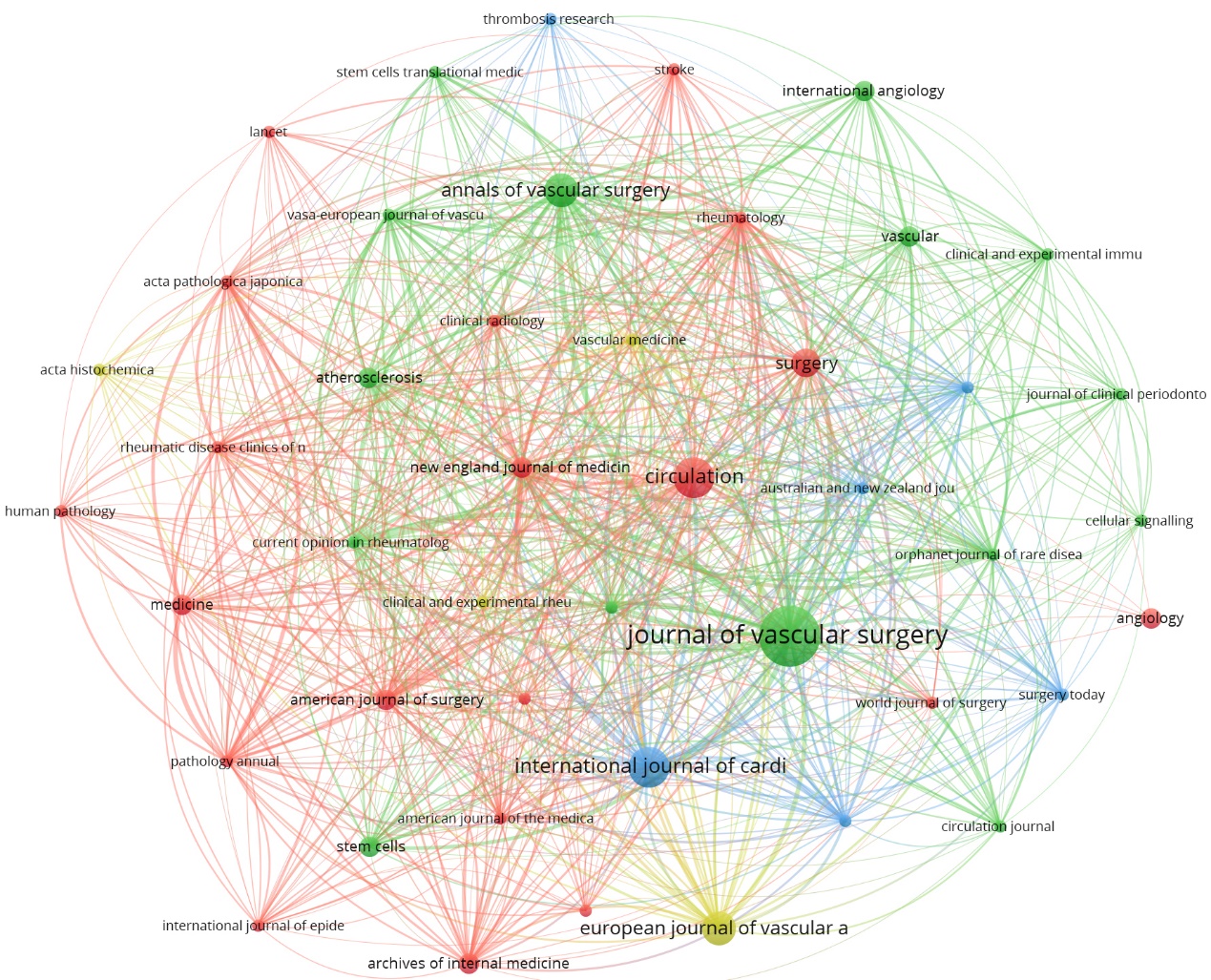
**Supplementary Fig. 4**  Bibliographic coupling analysis of journals


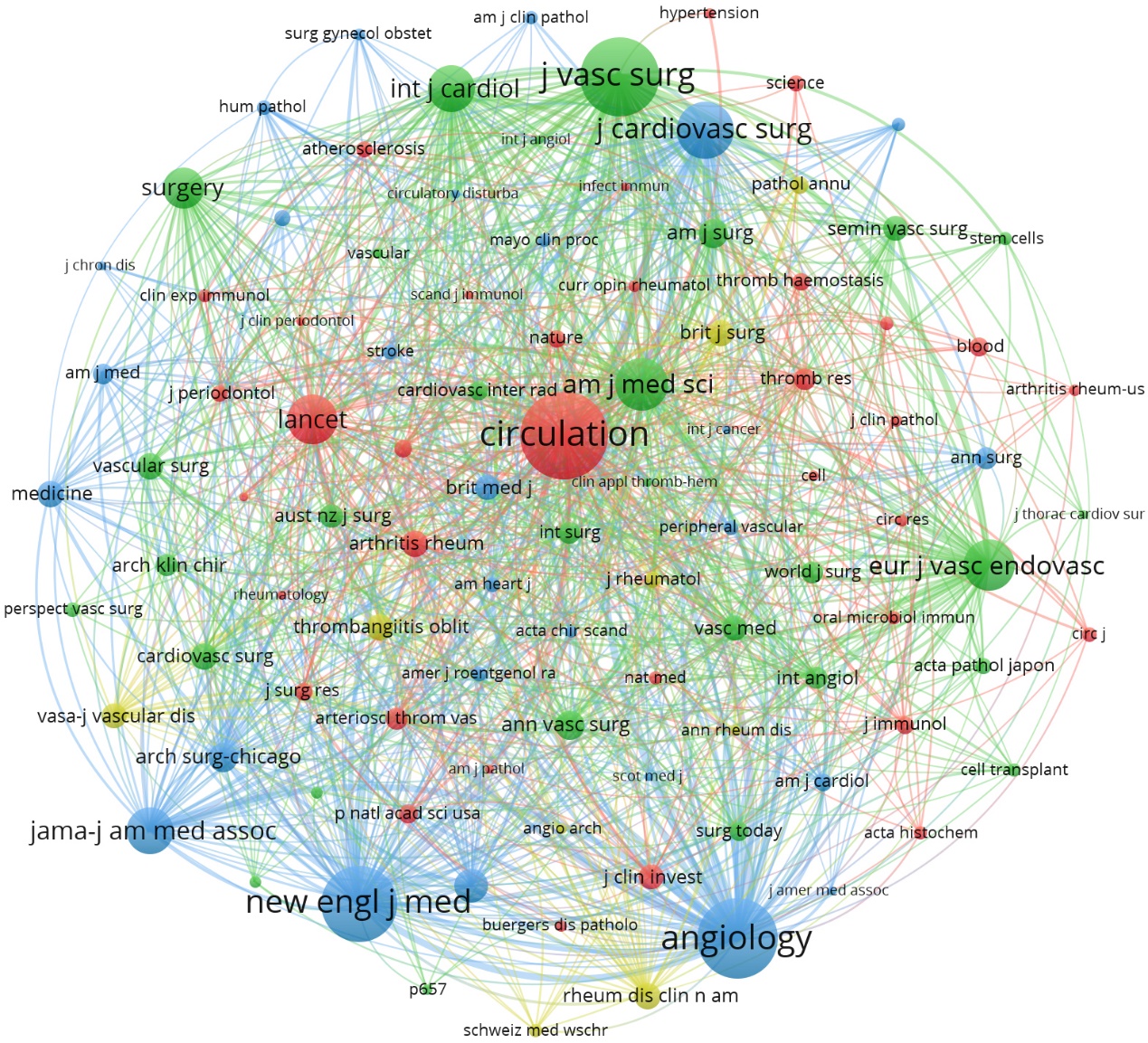
**Supplementary Fig. 5**  Co-citation analysis of highly cited journals in the references

**Supplementary Table 1** Top 10 most cited journals in the references

| Journal | Times counted | Total link strength | Impact Factor (2021) |
| --- | --- | --- | --- |
| Circulation | 116 | 1080 | 39.918 |
| Angiology | 103 | 1531 | 3.299 |
| Journal of Vascular Surgery | 99 | 1283 | 4.860 |
| New England Journal of Medicine | 94 | 1048 | 176.079 |
| Journal of Cardiovascular Surgery | 64 | 825 | 1.595 |
| American Journal of The Medical Sciences | 57 | 754 | 3.462 |
| European Journal of Vascular and Endovascular Surgery | 55 | 793 | 6.427 |
| Lancet | 52 | 602 | 202.731 |
| International Journal of Cardiology | 48 | 755 | 4.039 |
| JAMA | 48 | 613 | 157.335 |

**Supplementary Table 2** List of the top 100 cited articles

| No. | Title | Journal | Year | Citations |
| --- | --- | --- | --- | --- |
| 1 | Current concepts: thromboangiitis obliterans (Buerger's disease) | New England Journal of Medicine | 2000 | 347 |
| 2 | Treatment of thromboangiitis obliterans (Buerger's disease) by intramuscular gene transfer of vascular endothelial growth factor: Preliminary clinical results | Journal of Vascular Surgery | 1998 | 288 |
| 3 | Trial of iloprost versus aspirin treatment for critical limb ischemia of thromboangiitis-obliterans | Lancet | 1990 | 218 |
| 4 | Successful stem cell therapy using umbilical cord blood-derived multipotent stem cells for Buerger's disease and ischemic limb disease animal model | Stem Cells | 2006 | 189 |
| 5 | Diagnostic criteria of Buerger's disease | International Journal of Cardiology | 1998 | 187 |
| 6 | The changing clinical spectrum of thromboangiitis-obliterans (buergers-disease) | Circulation | 1990 | 177 |
| 7 | A critical evaluation of thromboangitis obliterans. The case against Buerger's disease. | The New England Journal of Medicine | 1960 | 168 |
| 8 | Cellular-sensitivity to collagen in thromboangiitis obliterans | New England Journal of Medicine | 1983 | 167 |
| 9 | Autologous bone-marrow mononuclear cell implantation for patients with Rutherford grade II-III thromboangiitis obliterans | Journal of Vascular Surgery | 2006 | 124 |
| 10 | Unblinded pilot study of autologous transplantation of bone marrow mononuclear cells in patients with thromboangiitis obliterans | Circulation | 2006 | 118 |
| 11 | Oral bacteria in the occluded arteries of patients with Buerger disease | Journal of Vascular Surgery | 2005 | 101 |
| 12 | Buerger's disease in the 21st century: diagnosis, clinical features, and therapy. | Seminars in Vascular Surgery | 2003 | 93 |
| 13 | Thromboangiitis obliterans and arteriosclerosis obliterans. Clinical and prognostic differences. | Annals of Internal Medicine | 1963 | 92 |
| 14 | Clinical and social consequences of Buerger disease | Journal of Vascular Surgery | 2004 | 88 |
| 15 | Antiendothelial cell antibodies in thromboangiitis obliterans | American Journal of the Medical Sciences | 1998 | 87 |
| 16 | Thromboangiitis obliterans (Buerger's disease) | Current Opinion in Rheumatology | 2006 | 86 |
| 17 | Cerebral thromboangiitis obliterans. | Medicine | 1957 | 85 |
| 18 | Buerger disease in the modern era | American Journal of Surgery | 1987 | 83 |
| 19 | Thromboangiitis obliterans or Buerger's disease: challenges for the rheumatologist | Rheumatology | 2007 | 78 |
| 20 | A point scoring system for the clinical diagnosis of Buerger's disease | European Journal of Vascular and Endovascular Surgery | 1996 | 76 |
| 21 | Immunohistochemical analysis of arterial wall cellular infiltration in Buerger's disease (endarteritis obliterans) | Journal of Vascular Surgery | 1999 | 76 |
| 22 | Association of HLA-A9 and HLA-B5 with Buerger's disease. | British Medical Journal | 1976 | 76 |
| 23 | Cogan's syndrome and systemic vascular disease. Analysis of pathologic features with reference to its relationship to thromboangiitis obliterans (Buerger). | Archives of Pathology | 1961 | 74 |
| 24 | Role of infrainguinal bypass in Buerger's disease: An eighteen-year experience | European Journal of Vascular and Endovascular Surgery | 1997 | 71 |
| 25 | The rise and fall and resurgence of thromboangiitis obliterans (buergers-disease) | Acta Pathologica Japonica | 1989 | 71 |
| 26 | Thromboangiitis-obliterans (buergers-disease) in women | Medicine | 1987 | 63 |
| 27 | Effect of autologous bone-marrow cell transplantation on ischemic ulcer in patients with Buerger's disease | Circulation Journal | 2007 | 60 |
| 28 | Impaired endothelium-dependent vasorelaxation in peripheral vasculature of patients with thromboangiitis obliterans (Buerger's disease) | Circulation | 1996 | 60 |
| 29 | Thromboangiitis obliterans (Buerger's disease) | Orphanet Journal of Rare Diseases | 2006 | 59 |
| 30 | What is buergers-disease | World Journal of Surgery | 1983 | 59 |
| 31 | Therapeutic angiogenesis in Buerger's disease (thromboangiitis obliterans) patients with critical limb ischemia by autologous transplantation of bone marrow mononuclear cells | Journal of Vascular Surgery | 2008 | 58 |
| 32 | Autoimmune mechanisms in thromboangiitis-obliterans (buergers-disease) - the role of tobacco antigen and the major histocompatibility complex | Surgery | 1992 | 58 |
| 33 | Diagnosis, pathology, and treatment of Buerger's disease. | Surgery | 1974 | 58 |
| 34 | A reconsideration of the pathogenesis of Buerger's disease. | American Journal of Clinical Pathology | 1958 | 56 |
| 35 | Thromboangiitis obliterans (buergers-disease) revisited | Pathology Annual | 1988 | 53 |
| 36 | Buerger's Disease | Annals of Vascular Surgery | 2012 | 51 |
| 37 | Diagnostic criteria and treatment of Buerger's disease: a review. | The International Journal of Lower Extremity Wounds | 2006 | 50 |
| 38 | Buergers-disease (thromboangiitis-obliterans) | Rheumatic Disease Clinics of North America | 1990 | 50 |
| 39 | Thromboangiitis Obliterans (Buerger's Disease)-Current Practices. | International Journal of Inflammation | 2013 | 49 |
| 40 | A review on thromboangiitis obliterans pathophysiology: thrombosis and angiitis, which is to blame? | Vascular | 2011 | 49 |
| 41 | Buerger's disease in Israel. | The American Journal of Medicine | 1965 | 49 |
| 42 | Buerger's disease: a review and update. | Seminars in Vascular Surgery | 1993 | 49 |
| 43 | Autoimmunological aspects of thromboangiitis obliterans (Buerger's disease). | Clinical Immunology and Immunopathology | 1978 | 48 |
| 44 | Buerger's disease: diagnosis and management. | Cardiovascular Surgery | 1993 | 47 |
| 45 | Thromboangiitis obliterans in the 21st century-A new face of disease | Atherosclerosis | 2009 | 47 |
| 46 | Immunobiologic analysis of arterial tissue in Buerger's disease | European Journal of Vascular and Endovascular Surgery | 2003 | 47 |
| 47 | Angiogenesis facilitated by autologous whole bone marrow stem cell transplantation for Buerger's disease | Stem Cells | 2006 | 46 |
| 48 | Surgical treatment of Buerger's disease. | Vascular | 2004 | 46 |
| 49 | Thromboangiitis-obliterans with eosinophilia (buergers-disease) of the temporal arteries | Human Pathology | 1988 | 45 |
| 50 | Oral iloprost in the treatment of thromboangiitis obliterans (Buerger's disease): A double-blind, randomised, placebo-controlled trial | European Journal of Vascular and Endovascular Surgery | 1998 | 44 |
| 51 | Abnormal plasma fibrin clot characteristics are associated with worse clinical outcome in patients with peripheral arterial disease and thromboangiitis obliterans | Atherosclerosis | 2011 | 44 |
| 52 | A randomized trial of intravenous iloprost (a stable prostacyclin analogue) versus lumbar sympathectomy in the management of Buerger's disease | International Angiology | 2006 | 44 |
| 53 | Expression of ICAM-1, VCAM-1, E-selectin and TNF-alpha on the endothelium of femoral and iliac arteries in thromboangiitis obliterans | Acta Histochemica | 2002 | 43 |
| 54 | Administration of Adult Human Bone Marrow-Derived, Cultured, Pooled, Allogeneic Mesenchymal Stromal Cells in Critical Limb Ischemia Due to Buerger's Disease: Phase II Study Report Suggests Clinical Efficacy | Stem Cells Translational Medicine | 2017 | 43 |
| 55 | Pathology and pathogenesis of Buerger's disease | International Journal of Cardiology | 1998 | 43 |
| 56 | Urinary cotinine measurement in patients with buergers-disease - effects of active and passive smoking on the disease process | Journal of Vascular Surgery | 1991 | 43 |
| 57 | Thromboangiitis obliterans. Clinico-angiographic correlations. | Archives of Surgery | 1964 | 43 |
| 58 | Implantable spinal cord stimulator to treat the ischemic manifestations of thromboangiitis obliterans (Buerger's disease) | Journal of Vascular Surgery | 1999 | 42 |
| 59 | The case for retention of the diagnostic category thromboangiitis obliterans. | Circulation | 1962 | 42 |
| 60 | Clinical Outcome After Extended Endovascular Recanalization in Buerger's Disease in 20 Consecutive Cases | Annals of Vascular Surgery | 2012 | 41 |
| 61 | Thromboangiitis obliterans (Buerger's disease). | Annals of Medicine and Surgery | 2016 | 39 |
| 62 | Natural-history of buergers disease | Journal of Cardiovascular Surgery | 1980 | 39 |
| 63 | Antiphospholipid antibodies in thromboangiitis obliterans | Vascular Medicine | 2002 | 39 |
| 64 | The role of epidural spinal cord stimulation in the treatment of Buerger's disease | Journal of Vascular Surgery | 2005 | 38 |
| 65 | Autoantibodies in thromboangiitis obliterans (buergers-disease) | Angiology | 1982 | 38 |
| 66 | Intestinal buergers-disease | Archives of Pathology & Laboratory Medicine | 1985 | 38 |
| 67 | Buerger's disease (thromboangiitis obliterans). | Annals of Vascular Surgery | 1991 | 38 |
| 68 | Decrease in prevalence of Buerger's disease in Japan | Surgery | 1998 | 37 |
| 69 | Vascular reconstruction in Buerger's disease: Is it feasible? | Surgery Today | 2002 | 37 |
| 70 | Cocaine and Buerger disease - Is there a pathogenetic association? | Archives of Internal Medicine | 2000 | 37 |
| 71 | Thromboangiitis obliterans: fact or fancy. | Circulation | 1961 | 37 |
| 72 | Immunological studies in thromboangitis obliterans (Buerger's disease). | The Journal of Surgical Research | 1979 | 36 |
| 73 | A case for cerebral thromboangiitis obliterans | Stroke | 1981 | 36 |
| 74 | Association of thromboangiitis obliterans with cigarette and bidi smoking in Bangladesh: a case-control study | International Journal of Epidemiology | 2000 | 35 |
| 75 | Activation of cytokines corroborate with development of inflammation and autoimmunity in thromboangiitis obliterans patients | Clinical and Experimental Immunology | 2012 | 34 |
| 76 | Buerger's disease in Hong Kong: A review of 89 cases | Australian and New Zealand Journal of Surgery | 1997 | 34 |
| 77 | The role of prothrombotic mutations in patients with Buerger's disease | Thrombosis Research | 2000 | 34 |
| 78 | Thromboangiitis obliterans. | British Heart Journal | 1966 | 34 |
| 79 | Intraarterial streptokinase as adjuvant therapy for complicated buergers-disease - early trials | International Surgery | 1993 | 33 |
| 80 | One of the most frequent vascular diseases in northeastern of Turkey: Thromboangiitis obliterans or Buerger's disease (experience with 344 cases) | International Journal of Cardiology | 2006 | 33 |
| 81 | Thromboangiitis obliterans associated with idiopathic hypereosinophilia | Archives of Internal Medicine | 1985 | 33 |
| 82 | Arteriographic findings in thromboangiitis obliterans with emphasis on femoropopliteal involvement. | The American Journal of Roentgenology, Radium Therapy, and Nuclear Medicine | 1970 | 33 |
| 83 | Recent views on Buerger's disease. | Journal of Clinical Pathology | 1969 | 32 |
| 84 | Thromboangiitis obliterans an update on Buerger's disease | Western Journal of Medicine | 1998 | 32 |
| 85 | Buerger's disease revisited. | The Surgical Clinics of North America | 1969 | 32 |
| 86 | Cytokine production in thromboangiitis obliterans patients: New evidence for an immune-mediated inflammatory disorder | Clinical and Experimental Rheumatology | 2005 | 31 |
| 87 | The IL-6/STAT3 pathway regulates adhesion molecules and cytoskeleton of endothelial cells in thromboangiitis obliterans | Cellular Signalling | 2018 | 31 |
| 88 | Thromboangiitis obliterans (Buerger's disease) | Vasa-european Journal of Vascular Medicine | 2014 | 31 |
| 89 | Transfection of human HGF plasmid DNA improves limb salvage in Buerger's disease patients with critical limb ischemia | International Angiology | 2011 | 31 |
| 90 | Buergers-disease (thromboangiitis obliterans) - an analysis of the arteriograms of 119 cases | Clinical Radiology | 1982 | 30 |
| 91 | Association between periodontitis and anti-cardiolipin antibodies in Buerger disease | Journal of Clinical Periodontology | 2009 | 29 |
| 92 | Long-term follow-up of thromboangiitis obliterans | Vasa-journal of Vascular Diseases | 1998 | 28 |
| 93 | Buerger's Disease (Thromboangiitis Obliterans)- Management by Ilizarov's Technique of Horizontal Distraction. A Retrospective Study of 60 Cases | Indian Journal of Surgery | 2011 | 28 |
| 94 | Buerger's disease in women. A report of a case and a review of the literature. | Angiology | 1973 | 28 |
| 95 | Intestinal manifestation of buergers-disease - case-report and literature-review | American Surgeon | 1981 | 28 |
| 96 | Elevated IgG titers to periodontal pathogens related to Buerger disease | International Journal of Cardiology | 2007 | 27 |
| 97 | Current pathological and clinical aspects of Buerger's disease in Japan | Annals of Vascular Surgery | 2006 | 27 |
| 98 | Current trends in thromboangiitis obliterans (Buerger's disease) in women | American Journal of Surgery | 1999 | 27 |
| 99 | The change in concept and surgical treatment on Buerger's disease - personal experience and review | International Journal of Cardiology | 1998 | 27 |
| 100 | Surgical treatment of Buerger's disease: experience with 216 patients. | Cardiovascular Surgery | 1993 | 12 |

**Supplementary Table 3** Visual representation of the research domains of original articles

| **Order** | **Treatment** | **Manifestations** | **Pathophysiology** | **Etiology** | **Diagnosis** | **Prognosis** | **Epidemiology** |
| --- | --- | --- | --- | --- | --- | --- | --- |
| 2 | Vascular endothelial growth factor |  |  |  |  |  |  |
| 3 | Coagulation |  |  |  |  |  |  |
| 4 | Stem cell therapy |  |  |  |  |  |  |
| 6 |  |  |  |  |  |  |  |
| 8 |  |  | Immune system |  |  |  |  |
| 9 | Stem cell therapy |  |  |  |  |  |  |
| 10 | Stem cell therapy |  |  |  |  |  |  |
| 11 |  |  |  |  |  |  |  |
| 13 |  |  |  |  |  |  |  |
| 14 |  |  |  |  |  |  |  |
| 15 |  |  | Autoimmune |  |  |  |  |
| 18 |  |  |  |  |  |  |  |
| 20 |  |  |  |  |  |  |  |
| 21 |  |  | Immune system |  |  |  |  |
| 22 |  |  | Genetics |  |  |  |  |
| 24 | Infrainguinal bypass |  |  |  |  |  |  |
| 26 |  |  |  |  |  |  |  |
| 27 | Stem cell therapy |  |  |  |  |  |  |
| 28 |  |  |  |  |  |  |  |
| 30 | Surgery |  |  |  |  |  |  |
| 31 | Stell cell therapy |  |  |  |  |  |  |
| 32 |  |  |  |  |  |  |  |
| 46 |  |  |  |  |  |  |  |
| 47 |  |  |  |  |  |  |  |
| 48 | Surgery |  |  |  |  |  |  |
| 50 | Oral iloprost |  |  |  |  |  |  |
| 51 |  |  | Coagulation |  |  |  |  |
| 52 | Drug and surgery |  |  |  |  |  |  |
| 53 |  |  | Endothelial dysfunction |  |  |  |  |
| 54 | Stell cell therapy |  |  |  |  |  |  |
| 56 |  |  |  |  |  |  |  |
| 57 | Endovascular surgery |  |  |  |  |  |  |
| 60 | Endovascular surgery |  |  |  |  |  |  |
| 62 |  |  |  |  |  |  |  |
| 63 |  |  | Autoimmunity |  |  |  |  |
| 64 | Spinal cord stimulation |  |  |  |  |  |  |
| 65 |  |  | Autoimmunity |  |  |  |  |
| 68 |  |  |  |  |  |  |  |
| 69 | Endovascular surgery |  |  |  |  |  |  |
| 72 |  |  | Autoimmunity |  |  |  |  |
| 74 |  |  |  |  |  |  |  |
| 75 |  |  | Inflammation and autoimmunity |  |  |  |  |
| 76 | Multiple |  |  |  |  |  |  |
| 77 |  |  | Genetics |  |  |  |  |
| 78 | Multiple |  | Multiple |  |  |  |  |
| 79 | Endovascular drug |  |  |  |  |  |  |
| 80 | Sympathectomy |  |  |  |  |  |  |
| 82 |  |  |  |  |  |  |  |
| 86 |  |  | Immune and inflammatory |  |  |  |  |
| 87 |  |  | Endothelial dysfunction |  |  |  |  |
| 89 | Human HGF plasmid DNA |  |  |  |  |  |  |
| 90 |  |  |  |  |  |  |  |
| 91 |  |  | Autoimmunity |  |  |  |  |
| 92 |  |  |  |  |  |  |  |
| 93 | Surgery |  |  |  |  |  |  |
| 96 |  |  | Immunity |  |  |  |  |
| 100 | Surgery |  |  |  |  |  |  |
| Counts | 24 | 4 | 17 | 11 | 4 | 5 | 3 |

**Supplementary Table 4** Visual representation of the research domains of reviews

| **Order** | **Treatment** | **Manifestations** | **Pathogenesis** | **Etiology** | **Diagnosis** | **Prognosis** | **Epidemiology** |
| --- | --- | --- | --- | --- | --- | --- | --- |
| 1 |  |  |  |  |  |  |  |
| 5 |  |  |  |  |  |  |  |
| 7 |  |  |  |  |  |  |  |
| 12 |  |  |  |  |  |  |  |
| 16 |  |  |  |  |  |  |  |
| 19 |  |  |  |  |  |  |  |
| 25 |  |  |  |  |  |  |  |
| 29 |  |  |  |  |  |  |  |
| 33 |  |  |  |  |  |  |  |
| 35 |  |  |  |  |  |  |  |
| 36 |  |  |  |  |  |  |  |
| 37 |  |  |  |  |  |  |  |
| 38 |  |  |  |  |  |  |  |
| 39 |  |  |  |  |  |  |  |
| 40 |  |  |  |  |  |  |  |
| 42 |  |  |  |  |  |  |  |
| 43 |  |  |  |  |  |  |  |
| 44 |  |  |  |  |  |  |  |
| 45 |  |  |  |  |  |  |  |
| 55 |  |  |  |  |  |  |  |
| 59 |  |  |  |  |  |  |  |
| 61 |  |  |  |  |  |  |  |
| 67 |  |  |  |  |  |  |  |
| 71 |  |  |  |  |  |  |  |
| 83 |  |  |  |  |  |  |  |
| 84 |  |  |  |  |  |  |  |
| 85 |  |  |  |  |  |  |  |
| 88 |  |  |  |  |  |  |  |
| 97 |  |  |  |  |  |  |  |
| 98 |  |  |  |  |  |  |  |
| 99 |  |  |  |  |  |  |  |
| Counts | 17 | 10 | 14 | 10 | 17 | 9 | 7 |
